# Generation of Heterotypic Primary Human Spheroids from Glioblastoma Resections and (Pre-)Clinical Applications

**DOI:** 10.3390/cells15111021

**Published:** 2026-06-02

**Authors:** Sophia Elisa Wiener, Jens Conrad, Saman Javid, Sophie Gieß, Max Jägersberg, Harald Krenzlin, Naureen Keric, Anne Régnier-Vigouroux, Carsten Geiß

**Affiliations:** 1 Institute for Developmental Biology and Neurobiology, Johannes Gutenberg University, 55128 Mainz, Germanysjavid@uni-mainz.de (S.J.); sgiess@uni-mainz.de (S.G.); vigouroux@uni-mainz.de (A.R.-V.); 2 Department of Neurosurgery, University Medical Center of the Johannes Gutenberg University Mainz, 55131 Mainz, Germany; jens.conrad@unimedizin-mainz.de (J.C.); max.jaegersberg@unimedizin-mainz.de (M.J.); harald.krenzlin@uksh.de (H.K.); naureen.keric@uksh.de (N.K.); 3 Department of Anesthesiology, University Medical Center of the Johannes Gutenberg University Mainz, 55131 Mainz, Germany

**Keywords:** glioblastoma, personalized medicine, brain tumors, spheroids, 3D-cultures, drug screening, invasion analysis, multicellularity

## Abstract

**Highlights:**

**What are the main findings?**

- Establishment of a protocol for robust generation of homogeneous primary spheroids from human glioblastoma specimens.
- Development of a (semi-)high-throughput, patient-specific screening platform.

**What are the implications of the main findings?**

- A simplified and reliable protocol based on commercially available products that can be readily implemented in both research and clinical laboratories.
- A scalable and versatile platform enabling patient-specific drug testing.

**Abstract:**

The rapid expansion of individualized treatment strategies necessitates advanced patient-specific screening platforms recapitulating tumor complexity. Here, we present such a platform preserving heterotypic cellular interactions and three-dimensional architecture which are both critical for predicting therapeutic responses. This cost-effective and versatile platform allows the rapid generation and functional testing of spheroids derived from primary human glioblastoma specimens. To maximize accessibility and ease of integration, we took advantage of commercially available kits to optimize the protocol of spheroid generation we had previously established. This enabled the robust, adaptable, and reproducible formation of homogeneous, multicellular spheroids within a few days. Spheroids generated from one patient specimen were structurally stable and showed a high degree of homogeneity over time. Immunohistochemical and flow cytometric analyses further revealed patient-specific cellular heterogeneity, underscoring the platform’s ability to preserve clinically relevant tumor features. Functionally, we demonstrate the applicability of this system for drug response profiling by assessing invasion dynamics following treatment with clinically relevant compounds. Collectively, our results establish a scalable and adaptable 3D screening platform that enables rapid, patient-specific phenotypic and functional analyses. This approach provides a powerful tool to complement existing clinical workflows and holds promise for improving the prediction of therapeutic responses in glioblastoma.

## 1. Introduction

Glioblastoma represents the most common type of malignant brain tumor with a poor survival rate [1]. Even novel treatment approaches, such as tumor-treating fields or immune checkpoint therapies, have failed to significantly extend patient survival [2,3]; thus, the standard of care still consists of tumor resection followed by chemo- and radiotherapy [3,4]. A major obstacle in cancer therapy is the intertumoral heterogeneity, which necessitates personalized treatment strategies. Designing such therapies requires processing of patient-derived material to identify suitable therapeutic targets (e.g., by sequencing or immunohistochemistry [IHC]). To subsequently ensure patient safety and improve success rates, selected treatments must be tested with an in vitro, patient-specific approach. This demands realistic and scalable cell culture systems using patient-derived material. Such cell culture systems must account for several factors that are often neglected in pre-clinical studies: the three-dimensional architecture of tumors, their cellular heterogeneity, and the presence of hypoxia. Strategies for immune cell-based therapies must be tested with culture systems including immune cells and enabling their direct contact with tumor cells. Additionally, other tumor-associated cells such as astrocytes should be included, as they contribute to tumor growth or therapy resistance [5]. While tumor tissue slice cultures preserve cellular heterogeneity and spatial distribution [6], they are of limited use in the case of drug-screening platforms due to the lack of homogeneous replicates. Other common strategies are based on the use of cell lines or induced pluripotent stem cells (iPSCs) [7], which can also be fused to brain organoids or bio-printed on a customizable matrix (please see [8] for a review on 3D glioblastoma models). These strategies share the same limitation that is that multicellular co-cultures cannot recapitulate the complex cellular heterogeneity found in tumors. Moreover, during the time of sub-culturing, these cell lines lose at least some of their original characteristics.

We therefore set ourselves the goal of designing a platform that would circumvent these problems. For that purpose, we established a multicellular 3D screening platform, based on spheroids generated with primary material from glioblastoma patients (referred to as primary tumor spheroids). To maximize possible applications and clinical utility, we sought to reduce the technical, methodological, and logistical requirements to a minimum. Our approach maintains the cellular heterogeneity of the original tumor, as illustrated by the composition in tumor cells and immune cells. Analysis of changes in cellular composition and cell distribution over time using flow cytometry and IHC indicates the robustness of the platform. Moreover, the system is highly scalable, yielding a large number of spheroids even from small tumor specimens, and utilizes 96-well plates that can be incubated under various oxygen conditions.

Beyond this biological characterization, we validated the pre-clinical relevance of the platform. For that purpose, we tested the response of several patient-derived glioblastoma spheroids to clinically relevant drug treatments by monitoring their invasion capacity. Our results underscore the utility of the platform for personalized drug screenings. Furthermore, they warrant the incorporation of in-depth assays and monitoring of further biological processes, such as cell viability and cell death, to screen and identify efficient patient-specific treatments. Finally, given the short spheroid generation time of maximum five days, individual patient-specific treatment approaches can be tested within a short time. We are convinced that, alongside routine diagnosis (e.g., mutations or methylation patterns), this platform will enhance the identification and validation of candidates for individualized treatment approaches, such as CAR-T cells, mRNA-vaccines, and novel drug combinations.

## 2. Materials and Methods

### 2.1. Glioblastoma Resections and Transport to the Laboratory

Tumor specimens were collected during surgical removal of tumor tissue from patients diagnosed with a high-grade brain tumor. When possible, a piece of brain tissue with a weight of approximately 1 g was transferred into a 15 mL tube containing 5 mL MACS^®^ Tissue Storage Solution (Miltenyi Biotec, Bergisch Gladbach, Germany). Samples were collected within 1 h after removal or otherwise stored for a maximum of 4 h at 4 °C until pickup and immediately transferred to the laboratory. Only specimens with a weight of ≥100 mg and with pathological confirmation of glioblastoma diagnosis were included in the study. Please find information about patient characteristics in Table S1.

### 2.2. Final Protocol for the Preparation of Single Cell Suspension of Glioblastoma Specimen

The protocol is based on the Brain Tumor Dissociation Kit (Miltenyi Biotec). In brief, the weight of tumor resection was determined. The resection was cut into small pieces (approx. 3 × 3 mm), and a maximum of 600 mg was used per gentleMACS™ C tube (Miltenyi Biotec). After addition of buffers X and Y, enzymes A and N, and 8 µL of collagenase II (100 U/µL, Sigma-Aldrich, St. Louis, MO, USA) per tube, C tubes were placed on a gentleMACS™ Dissociator (Miltenyi Biotec) running program h_tumor_02. After termination of the program, tubes were detached and incubated for 15 min at 37 °C under slow, continuous rotation using a MACSmix™ Tube Rotator (Miltenyi Biotec). Afterwards, tubes were again placed on the dissociator and the program h_tumor_03 was started. After termination of the program, samples were incubated for another 10 min at 37 °C under slow, continuous rotation. A final dissociation step using the program m_brain_01 was performed and 15 mL ice-cold HBSS (Gibco, Waltham, MA, USA) was added to the solution. The cell suspension was filtered using 70 and 30 μm cell strainers and cells were pelleted by centrifugation (400× *g*, 5 min, 4 °C). The supernatant was aspired and cells were resuspended in 10 mL ice-cold erythrocyte lysis buffer (0.1 mM EDTA, 10 mM KHCO_3_, 0.155 M NH_4_Cl). After 5 min of incubation on ice, cells were centrifuged (5 min, 400× *g*, 4 °C) and the supernatant was aspirated. The cell pellet was resuspended in 6.2 mL ice-cold PBS (Gibco) and 1.8 mL ice-cold debris removal solution (Miltenyi Biotec) was added and mixed by pipetting. The cell suspension was gently overlayed with 4 mL ice-cold PBS. After centrifugation (3000× *g*, 10 min, 4 °C; slow break and slow acceleration), the two upper phases were aspirated, cells were resuspended in 15 mL ice-cold PBS and centrifuged (1000× *g*, 10 min, 4 °C). The supernatant was removed and cells were resuspended in ice-cold GB-cDMEM (DMEM [Gibco], 10% heat-inactivated FBS [Sigma-Aldrich], 2 mM L-Glutamine [Gibco], 10 μg/mL Gentamicin [Carl Roth, Karlsruhe, Germany], and 1 mM CaCl_2_, 1 mM MgCl_2_) for immediate seeding, as described in Section 2.3.

### 2.3. Cell Seeding and Monitoring of Spheroid Formation

Cells were counted using a Countess 3 cell counter (Invitrogen, Carlsbad, CA, USA) with the exclusion of dead cells using trypan blue (Gibco). Living cells were resuspended in GB-cDMEM and 100 µL of the cell suspension was distributed per well into a BIOFLOAT™ 96-well plate (Sarstedt, Nümbrecht, Germany). Afterwards, cells were incubated under standard conditions (37 °C, 5% CO_2_, 90% RH) and monitored using a Cellcyte X (ECHO, San Diego, CA USA) automated microscope. Images were acquired at the indicated timepoints to determine spheroid area.

### 2.4. Flow Cytometry Analysis

Spheroids were collected at the respective time points and transferred into a 1.5 mL tube. Spheroids were washed by adding 1 mL HBSS. After settlement, the supernatant was removed and replaced by 1 mL digestion mix (HBSS containing 0.5 U/mL Liberase TL [Sigma-Aldrich] and 0.1 mg/mL DNase I [Roche, Mannheim, Germany]). Cells were incubated for 30 min at 37 °C. If spheroids were not fully dissociated, dissociation was supported mechanically by pipetting, and incubation was prolonged for another 15 min. Afterwards, cells were collected by centrifugation (400× *g*, 5 min, RT), the supernatant was removed and cells were fixed in 50% Fixation buffer (BioLegend, San Diego, CA, USA) for 10 min on ice. After centrifugation (400× *g*, 5 min, RT), cells were washed with staining buffer (0.5% BSA [Roche], 2 mM EDTA [Carl Roth], 0.1% NaN_3_ [Carl Roth]). After centrifugation (400× *g*, 5 min, RT), cells were permeabilized by washing two times with Intracellular Staining Permeabilization Wash Buffer (BioLegend, San Diego, CA, USA). Afterwards, cells were resuspended in 100 µL Intracellular Staining Permeabilization Wash Buffer and 2 µL FcR Blocking Reagent (Miltenyi Biotec) was added and incubated for 5 min at RT. Antibodies were added according to Table 1 and incubated for 15 min at RT. Cells were washed two times using staining buffer and analyzed using an Attune NxT flow cytometer (Invitrogen).

### 2.5. Spheroid Embedding, Monitoring, and Invasion Analysis

Primary tumor spheroids were embedded in a collagen matrix to assess invasive capacity. All steps for matrix preparation were performed on ice to prevent premature collagen polymerization. A total of 400 µL collagen mix (MEM [Gibco] containing 0.02 M NaOH and 2.2 mg/mL bovine collagen [CellSystems, Troisdorf, Germany] were added in each well of a 24-well plate (Sarstedt) and incubated at 37 °C for 2 min to allow initial polymerization. Afterwards, spheroids were carefully implanted in the middle of the well, and on the top of the collagen matrix with minimal medium carry-over. After 2 h incubation at 37 °C, the collagen was completely polymerized and 400 µL cDMEM (DMEM supplemented with 10% heat-inactivated FBS, 2 mM L-Glutamine, 10 μg/mL Gentamicin) containing drugs (Temozolomide, Lomustine, Regorafenib, Veliparib [Adooq Bioscience, Irvine, CA, USA]) or vehicle was added. Brightfield images were acquired using a Leica DM IL LED Fluo fluorescence microscope (Leica, Wetzlar, Germany) with 5× magnification at day 0 and other indicated time points.

Image analysis was performed using CellProfiler (version 4.2.8.) [9] to measure object size, shape, intensity, and texture. A standardized analysis pipeline was applied to all images. Briefly, brightfield images were first converted to gray using the ColorToGray action, with the conversion method “combine” to extract overall intensity information. Uneven background illumination was corrected using CorrectIlluminationCalculate, whereby the illumination function was estimated from the background, rescaled, and calculated individually for each image without smoothing or retention of averaged or dilated images. The resulting illumination function was applied using CorrectIlluminationApply by division, with output values constrained to a range of 0 to 1. The corrected gray images were converted back to a color format using GrayToColor with intensity rescaling to ensure compatibility with subsequent processing steps. Images were then inverted by action InvertForPrinting so the spheroid core and invasive area appeared as dark objects on a bright background. Afterwards, images were reconverted to grayscale using ColorToGray (conversion method “combine”). Spheroid core and invasive cells were identified using IdentifyPrimaryObjects. Thresholding was performed using a global threshold strategy with the Otsu method and logarithmic transformation prior to thresholding, enabling separation of three intensity-based classes corresponding to spheroid core, invasive area, and background. No declumping lines between touching objects were applied, and holes within identified objects were filled. For quantitative analysis, the spheroid core and invasive area were combined and treated as the primary object, while the background was excluded. Object identification proceeded only when an excessive number of objects was detected, ensuring robust and consistent segmentation. Quantitative analysis was performed using MeasureImageAreaOccupied to determine the total area occupied by the spheroid core and invasive region. For visualization, object outlines were superimposed onto the original images using OverlayOutlines. For data analysis, the occupied area of each spheroid was normalized to its respective area on day 0 and is therefore representing individual spheroid area relative to day 0.

### 2.6. Immunofluorescence Staining of Spheroid Cryosections

Primary tumor spheroids generated as described in Section 2.3. were washed with cold PBS, embedded in optimal cutting temperature (OCT) compound (Sakura Finetek, Alphen aan den Rijn, The Netherlands), and frozen at −80 °C. Cryosections (6 µm) were mounted on Superfrost Plus microscope slides (Epredia, Portsmouth, NH, USA). Sections were air-dried for 5 min and fixed with Fixation buffer for 10 min at RT. After washing three times with ice-cold PBS (10 min each), sections were permeabilized with 0.2% Triton X-100 in PBS for 10 min at RT, followed by an additional PBS wash. For CD45RO and CD3ε staining, the permeabilization step was omitted to preserve membrane-associated epitopes. Non-specific binding was blocked using blocking buffer (5% goat serum [biowest, Nuaillé, France], 1% BSA in PBS) for 60 min at RT.

Sections were incubated overnight at 4 °C with primary antibodies (see Table 2) diluted in blocking buffer and combined as follows: mouse anti-GFAP combined with rabbit anti-IBA1/AIF-1, or mouse anti-CD45RO combined with rabbit anti-CD3ε. Following three washes with ice-cold PBS, sections were incubated for 60 min at RT with fluorophore-conjugated secondary antibodies. Negative control sections were included for each staining set by omitting primary antibodies while keeping all other steps identical. Slides were washed and covered with Fluorescence Mounting Medium (Agilent, Santa Clara, CA, USA) containing 1 µg/mL Hoechst 33342 (Life Technologies, Carlsbad, CA, USA).

### 2.7. Image Analysis and Quantification

Images were acquired using a Leica DM IL LED Fluo fluorescence microscope equipped with a Leica DFC450 C digital camera. Each slide contained a single spheroid, which was imaged in its entirety within one field of view. Exposure settings were optimized using negative control sections to minimize background fluorescence and were kept constant across all samples.

Image analysis was performed using ImageJ software (version 1.54p). Total cell numbers were determined from Hoechst 33342-stained nuclei following conversion to 8-bit grayscale, local thresholding using the Phansalkar method (radius = 15), watershed segmentation, and particle analysis with a size threshold of ≥80 pixels and a circularity range of 0.30–1.00. Nuclear Regions of Interest (ROIs) were generated, and total cell counts were extracted. To account for marker-specific subcellular localization, nuclear ROIs were isotropically expanded prior to marker quantification. ROI expansion distances were empirically determined based on marker-specific localization and optimized to maximize capture of true signal while minimizing background, using negative control sections. Accordingly, ROIs were expanded by 8 pixels for cytoplasmic markers (GFAP and IBA1) and by 25 pixels for membrane-associated markers (CD45RO and CD3ε). Percentages of marker positive area relative to the expanded ROI area (Area%) were quantified using thresholds defined based on negative controls and applied uniformly across all samples. Since CD3+ cells were commonly rare and we observed a high number of artifacts, we considered only cells double positive for CD3 and CD45 as CD3+ cells.

### 2.8. LDH Release Assay

Release of lactate dehydrogenase (LDH) was measured using CytoTox 96^®^ Non-Radioactive Cytotoxicity Assay (Promega, Madison, WI, USA) following manufacturer’s instructions. In brief, 50 µL supernatant was collected at indicated time points and centrifuged (400× *g*, 5 min, RT). Afterwards, an equal volume of CytoTox 96^®^ Reagent was added and samples were incubated for 30 min at RT in the dark. The reaction was stopped by adding 50 µL stop solution and absorbance was recorded at 490 nm using a GloMax plate reader (Promega).

### 2.9. Formulas and Statistics

Spheroid volume was calculated using the measured area (A) and the formula for sphere volume. Please note that the shape of a perfect sphere was assumed to calculate the volume:

$$spheroid\;volume=\frac{4}{3}\pi *{\left(\sqrt{\frac{A}{\pi }}\right)}^{3}$$

Spheroid roundness was calculated using measured spheroid area (A) and perimeter (U):

$$spheroid\;roundness=\;\frac{2\pi *\sqrt{\frac{A}{\pi }}}{U}$$

## 3. Results

### 3.1. Identification of Optimal Conditions for Generation of Spheroids

**Figure 1:**
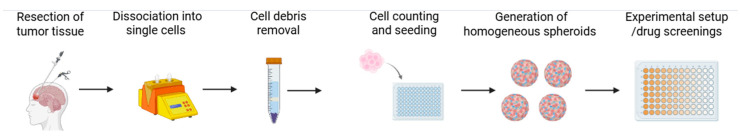
Schematic illustration of the protocol workflow. After tumor resection, glioblastoma specimens are dissociated into single cells, purified, counted, and seeded in 96-well ultra-low-attachment plates. Homogenous multicellular spheroids form in four to five days and are ready to be used for subsequent experiments. Created in BioRender. Geiß, C. (2026) https://BioRender.com/cg3udbm (accessed on 30 March 2026).

As a starting point, we used the protocol we have established for generating spheroids with rodent (murine and rat) and human glioblastoma cell lines [10,11,12]. Based on our knowledge in generating spheroids consisting of a single tumor cell line or a mixture of tumor cells and microglia or macrophages, we systematically tested several factors with the final goal of obtaining homogeneous round, compact, stable and viable primary tumor spheroids in a short period of time. This systematic analysis was conducted with a fresh glioblastoma specimen from, in total, more than 50 patients and is described in Section 3.1 and Section 3.2. This resulted in the final protocol illustrated in Figure 1, which was used to generate the spheroids for subsequent analysis.

**Figure 2:**
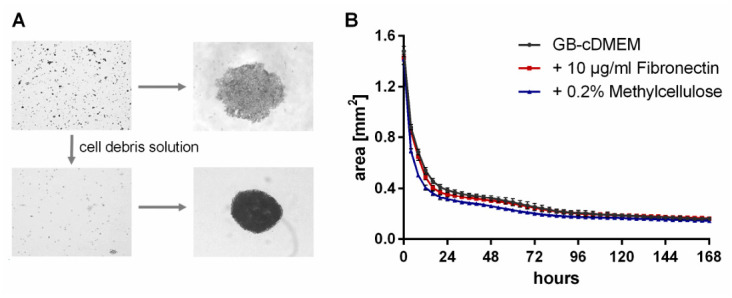
Formation of spheroids is improved by the removal of cellular debris, but not by the addition of fibronectin or methylcellulose. (**A**) Top: single cell solution and resultant spheroids after 96 h without debris removal; bottom: single cell solution and resultant spheroids after 96 h with debris removal. (**B**) Spheroid areas up to 168 h after cell seeding using GB-cDMEM with or without the addition of 10 µg/mL fibronectin or 0.2% methylcellulose. Shown are mean values ± SD; n = 6 technical replicates.

The successful generation of stable spheroids depends on various conditions. Before starting the enzymatic dissociation steps, blood vessels and necrotic tissue were carefully removed. Since collagen levels are often increased in gliomas [13], we added collagenase during enzymatic digestion to increase efficacy and reduce the number of cell clumps (for details see Section 2.2). In some cases, the single cell suspension obtained after the enzymatic digestion contained a lot of debris which inhibited proper spheroid formation. We therefore included a purification step using debris removal solution, which led to a small reduction in total cell number, but dramatically improved the quality of the single cell suspension, facilitating spheroid formation (Figure 2A). This purification step was the most successful in improving the quality of the cell suspension. Of note: the use of myelin removal beads did not lead to any improvement. Studies have reported that addition of methylcellulose (ThermoFisher, Waltham, MA, USA) or fibronectin (Corning, Corning, NY, USA) to the cell suspension could have a supportive effect on the capacity of single cells to form a spheroid [14,15,16], but in our hands, none of these additives were necessary nor improved or accelerated spheroid formation (Figure 2B). Furthermore, we tested various growth media based on DMEM, DMEM-F12, or Neurobasal medium, supplemented with different combinations of 5–10% FBS, growth factors (EGF, FGF [PeproTech, Hamburg, Germany]), and supplements (B-27, N-2 [Gibco]). We did not observe any benefit using the more complex DMEM-F12 (Gibco) or Neurobasal media (Gibco), compared to our standard growth medium (GB-cDMEM), consisting of DMEM supplemented with FBS, glutamine and antibiotics. Of note: the addition of 1 mM CaCl_2_ and 1 mM MgCl_2_ accelerated cell–cell binding in some specimens and was therefore routinely added to the media.

### 3.2. Characterization of Cell Number on the Formation Capability and Size of Spheroids

**Figure 3:**
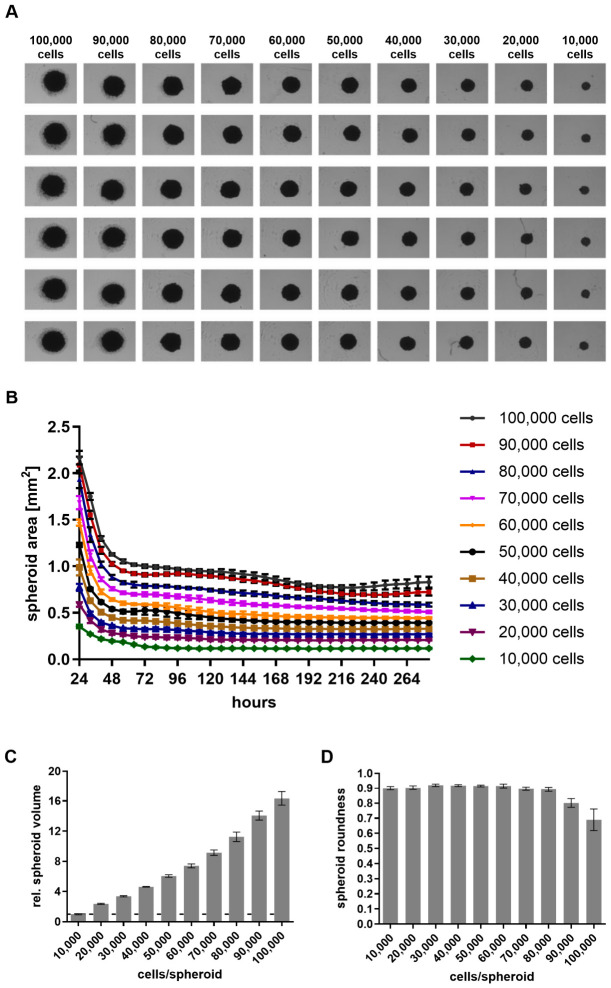
Increasing cell numbers does not interfere with spheroid formation and results in the formation of larger spheroids, but it affects spheroid roundness. (**A**) Representative images of spheroids generated using 10,000–100,000 single cells 280 h after seeding. (**B**–**D**) Quantitative analysis of spheroids shown in (**A**). (**B**) Time-dependent compaction of spheroids. (**C**) Spheroid volume relative to 10,000 cells/spheroid. (**D**) Roundness of the spheroids. Data represented as mean values ± SD; n = 6 technical replicates.

The last and highly relevant factor to consider for the generation of spheroids mimicking genuine tumors is the number of cells to be seeded, which determines the size of the spheroid. Indeed, oxygen and nutrient gradients depend on spheroid size [17]. Moreover, too few or too many cells could interfere with their capacity to form spheroids or with spheroid stability. To identify suitable cell numbers, spheroids were generated using different cell numbers starting from 10,000 up to 100,000 cells (Figure 3 and Figures S1). Live imaging monitoring showed that, irrespective of the starting cell number, cell compaction and generation of spheroids followed the same trend (Figure 3A,B). However, the time required to reach stable spheroid areas increased with cell number (Figure 3B; e.g., compare spheroids with 20,000 cells/120 h versus 80,000 cells/264 h). As expected, the calculated volume of spheroids increased with cell number (Figure 3C), but spheroid’s roundness decreased with high cell numbers, suggesting a lack of stability (Figure 3D). Based on these observations, we selected 20,000 cells as the seeding number for further experiments to maximize the number of spheroids that can be obtained from the limited amount of primary material. Furthermore, spheroid formation time was set to 96 h to 120 h.

### 3.3. Primary Tumor Spheroids Generated from Individual Tumors Show a Homogenous Compaction Pattern and Spheroid Size

Having established the conditions for spheroid generation and maintenance in medium over a few days (Figure 3), we next aimed to examine their behavior in a 3D collagen matrix. Indeed, most drug-screening platforms use spheroids kept in solution. However, implantation of spheroids in a matrix enables the setup of a complex 3D environment that is highly relevant for pre-clinical testing. Indeed, it provides the experimenter with the possibility to evaluate the effects of drugs by monitoring changes in size of the spheroid and cell capacity to migrate into the matrix. These parameters indirectly reflect cell viability, composition of the spheroids and differentiation state.

**Figure 4:**
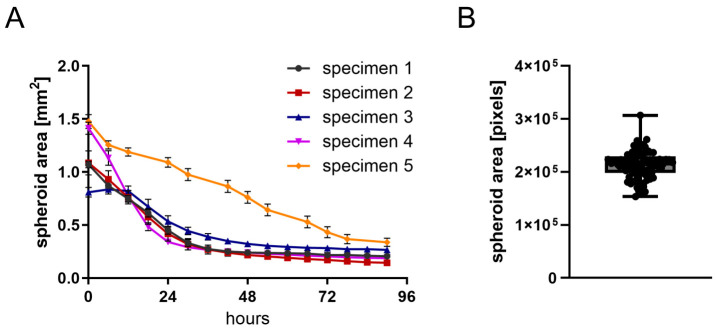
Compaction profiles and consistency of spheroids generated from glioblastoma specimens. (**A**) Analysis of spheroids area derived from five different specimens. Each well of a 96-well plate was filled with 20,000 cells extracted from primary tumor tissue and spheroid area was monitored for up to 90 h. Graph shows mean area +/− standard deviation. (**B**) Boxplot of spheroid area of 80 spheroids derived from the same specimen directly after embedding in collagen; dots represent individual spheroids.

Primary tumor spheroids were generated from five different donors. Comparable to our previous observation (Figure 3B), most specimens showed a similar pattern characterized by a fast compaction at the beginning, which is slowed down after 48 h (Figure 4A). Only one specimen showed slower compaction behavior, reaching a stable size 72 h after seeding. We next analyzed the size of multiple spheroids generated from one patient’s resection in order to test their uniformity in a 3D environment. To that end, spheroids were embedded in a collagen matrix and immediately imaged. As shown in Figure 4B, after their transfer into the collagen matrix, the spheroids displayed a homogenous phenotype, characterized by their uniform size.

### 3.4. Cellular Spheroid Composition and Distribution of Tumor and Immune Cells over Time

**Figure 5:**
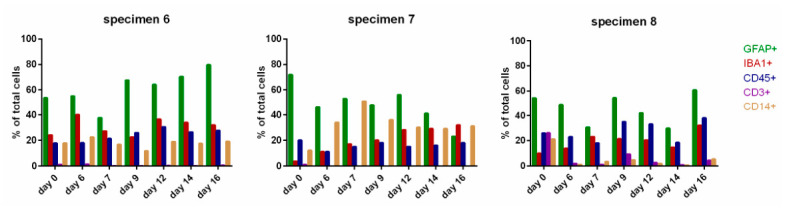
Analysis of the cellular composition of spheroids up to 16 days after spheroid formation by flow cytometry. Single cell solutions were obtained from material derived from three different patients’ resections (specimens 6, 7, 8) by enzymatic dissociation of tumor specimens (day 0) or of tumor-derived spheroids (days 6–16).

To further analyze the homogeneity of spheroids generated with one patient’s material, and their stability in culture over a given period of time, we examined their content in tumor cells and immune cells. We analyzed aliquots of the single cell suspension prepared on the day of tumor resection (day 0) and single cell suspensions from spheroids harvested at different times during the formation period. To evaluate the cellular composition of the spheroids, we monitored GFAP (Glial Fibrillary Associated Protein) as a marker for tumor cells and (tumor-associated) astrocytes, CD45 (leucocyte common antigen) as a specific marker of immune cells (mainly macrophages and T cells), CD14 as a specific marker of monocytes, IBA1 as a specific marker of macrophages and microglia, and CD3 as a specific marker of T cells. Three exemplary cases are shown in Figure 5. Data for day 0 (immediately after tissue dissociation) are consistent with previously published data [18] and reflect the intertumoral heterogeneity of glioblastoma specimen. GFAP+ cells represented half or more than the total number of cells analyzed, the other half or less being myeloid cells. CD3+ cells were hardly detectable. Our longitudinal analysis revealed that the number of GFAP+ cells decreased from day 0 to day 6 to a stable level until it decreased again at day 14 and 16. This decrease most likely indicates a loss of these cells, as indicated by the reduction in spheroid size with time (Figure 3B). The number of CD45+, IBA1+, CD14+ and, whenever detectable, CD3+ cells were stable between day 6 and day 16, indicating a stable population of these cells in the spheroids.

### 3.5. Regional Localization of Different Cell Types in the Spheroids

**Figure 6:**
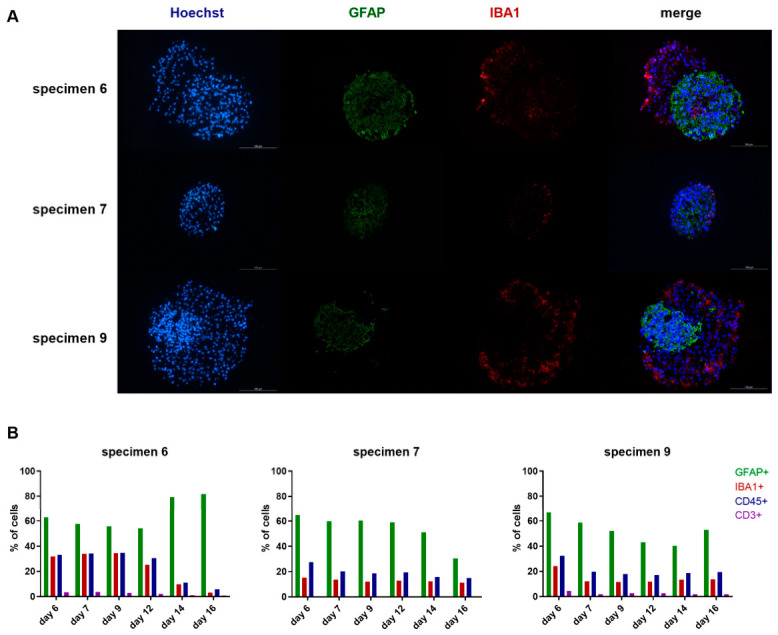
Analysis of cellular composition and cell distribution in primary tumor spheroids. (**A**) Representative images of three different samples (specimens 6, 7, 9) 12–14 days after cell seeding showing cell nuclei (Hoechst), GFAP+ cells, IBA+ cells, and a merged image. Staining intensities were partially increased to improve image quality; scale bar = 100 µm. (**B**) Quantification of GFAP-, IBA1-, CD45-, and CD3-positive cells of the same specimen.

Flow cytometry gives insights into the overall cellular composition of the spheroids, but it does not reflect the local distribution of the cells. Therefore, we used immunohistochemistry to analyze the distribution of GFAP+, IBA1+, CD45+, and CD3+ cells after spheroid formation (Figure 6A and Figures S1). Quantification of different cell types (Figure 6B) was consistent with the flow cytometry analysis (Figure 5), although we observed some differences which could be explained by the different methodology and the non-equal distribution of cell types in the spheroids (specimens 6 and 7 were used for both analyses). Especially IBA1+ cells showed a high accumulation at the edges of the spheroid. Moreover, in some specimens, a clustering of GFAP+ cells was observed and they clearly separated from CD45+ cells over time (e.g., specimen 6, see Figure 6 and Figures S1).

### 3.6. Characterization of Patient-Specific Drug Responses Using Primary Glioblastoma Spheroids

**Figure 7:**
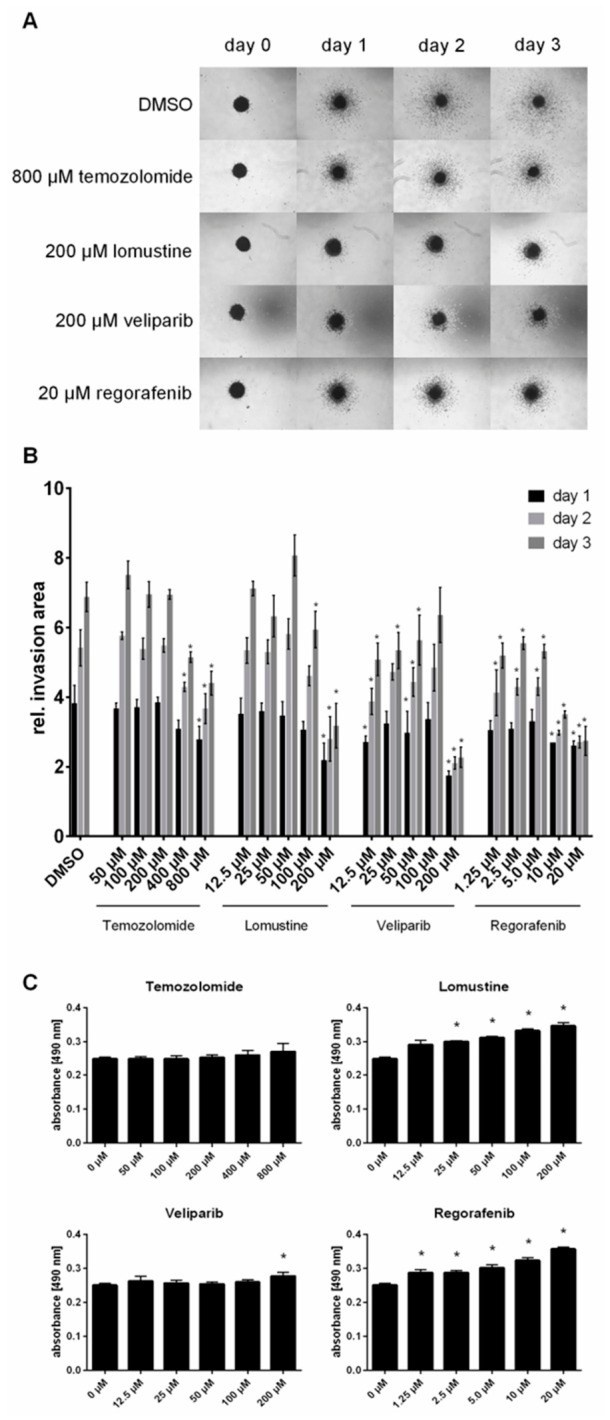
Titration of clinically relevant drugs using spheroid invasion analysis. Spheroids were generated with one patient’s resection. (**A**) Representative images of spheroids embedded in collagen and treated with increasing doses of temozolomide, lomustine, veliparib, and regorafenib. (**B**) Quantitative analysis of invasion of the spheroids shown in (**A**). n = 4 spheroids; two-way Anova with Bonferroni’s multiple comparisons test. (**C**) LDH assay of the spheroid supernatants after three days of treatment. n = 4 spheroids; two-tailored *t*-test. * = significant changes with *p* < 0.05.

Our overall aim was to establish a protocol enabling patient-specific drug screenings in a multicellular 3D model. To validate the developed protocol, multiple spheroids generated from one patient’s resection were embedded in a collagen matrix and treated with temozolomide and other clinically relevant drugs used in glioblastoma therapy [19,20,21,22,23]. The invasion capacity of the treated spheroids was monitored over three days by measuring cell infiltration into the collagen matrix (Figure 7A,B). The invasion capacity of the spheroids was largely unaffected by temozolomide. This result is consistent with the patient’s negative *MGMT* methylation status, as resistance to temozolomide typically correlates with the methylation status of the O6-methylguanine-DNA methyl-transferase (MGMT) gene promoter [24].

The other tested drugs reduced or suppressed the invasion capacity, especially at the highest doses tested. Reduced invasion capacity can result from a decrease in proliferation, migration capacity, or cell death. We tested the latter by analyzing LDH release in the spheroid supernatants after three days of treatment (Figure 7C). We observed significant dose-dependent increases for lomustine- and regorafenib-treated spheroids, suggesting that cell death contributed to the reduced invasion. For veliparib, a significant increase was only detectable with the highest tested dose, whereas for temozolomide, only a slight increase was observed.

**Figure 8:**
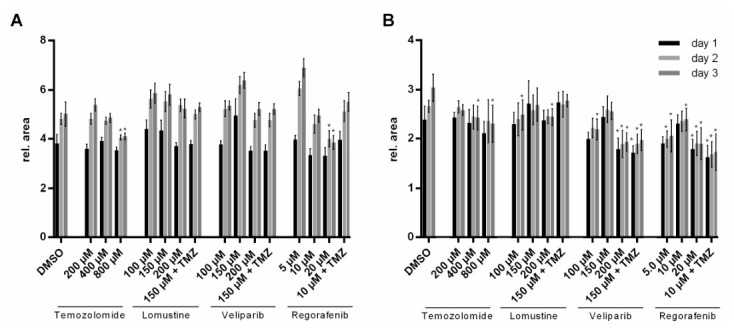
Invasion analysis of primary tumor spheroids treated with temozolomide, lomustine, veliparib, and regorafenib alone or in combination with temozolomide. Spheroids were treated with indicated drugs and doses alone or in combination with 400 µM temozolomide (+TMZ) and monitored for three days. (**A**) Specimen was tested negative for *MGMT* methylation. (**B**) Specimen was tested positive for *MGMT* methylation. n = 5 spheroids; two-way Anova with Bonferroni’s multiple comparisons test. * = significant changes with *p* < 0.05.

Another possible use of the platform is the testing of drug combinations. As temozolomide is the commonly used first-line treatment in glioblastoma therapy, we analyzed its combinations with each of the other tested drugs (Figure 7) and monitored the invasion capacity of primary tumor spheroids. For these proof-of-principle experiments, we used spheroids generated from a tumor resection tested negatively for *MGMT* methylation (Figure 8A) and spheroids generated from a tumor resection tested positively for *MGMT* methylation (Figure 8B). Untreated spheroids (DMSO control condition) from both specimens invaded the matrix, though at different rates, reflecting patient-specific characteristics. As expected, spheroids from the negative *MGMT* methylation resection were more resistant to temozolomide (Figure 8A), and combining temozolomide with any of the other drugs was fully inefficient. This was not the case with the spheroids from the resection with positive *MGMT* methylation (Figure 8B). It is worth noting that the observed effects on TMZ efficacy might reflect the cellular composition in addition to MGMT expression levels. Indeed, combining temozolomide with veliparib or regorafenib impaired invasion capacity, identifying these as potentially effective clinical combinations.

## 4. Discussion

In this study, we describe a protocol suitable for the generation of multiple homogeneous spheroids from glioblastoma specimens. Our protocol relies on standard devices and reagents without the need for expensive additives or media. Spheroids derived from a single patient’s resection exhibit uniform compaction behavior and consistent treatment responses, and remain stable for several days. Moreover, spheroid size is proportional to the seeded cell number and is therefore adjustable. Importantly, standardization of the protocol does not compromise patient-specific characteristics, as spheroids generated from different patients show different cellular composition, invasion capacity, and response to drugs. These advantages build a strong basis for (semi-)high-throughput spheroid generation and patient-specific drug testing. A particular strength of the platform is the abundance of tumor-associated immune cells, which opens opportunities for investigating immune cell-based therapies.

Nevertheless, successful spheroid generation strongly depends on the quality of the resected tumor material. We observed limited spheroid compaction if the tumor resections contained a high proportion of (most likely) non-tumor tissue, resulting in a low ratio of tumor cells to healthy cells. A high content of myelin will also interfere with spheroid compaction by blocking cell–cell interactions, a problem which can be circumvented—to a certain degree—by gradient centrifugation. Tumor specimens with extensive necrosis, for example due to ultrasonic aspiration, also pose challenges. We therefore recommend removing necrotic areas and blood vessels as thoroughly as possible prior to enzymatic dissociation to improve cell yield and prevent clogging of cell strainers.

In terms of yield, we obtained approximately 2 × 10^6^ cells per 100 mg of tumor tissue, which accounts for 100 spheroids (if 20,000 cells/spheroid are used). Based on our experience, we do not recommend starting with less than 100 mg of resected material.

The primary aim of this study was to establish a 3D platform that enables rapid, patient-specific drug screening following tumor resection and to provide a standardized protocol for the clinical and scientific community. While this objective was achieved, several limitations should be acknowledged. First, we observed a decline in GFAP-positive cells accompanied by a reduction in spheroid size approximately two weeks after tumor resection, indicating limited long-term stability of the model. Regular medium exchange and the incorporation of cell type-specific supplements may help mitigate these effects and improve the viability of distinct cell populations, including astrocytes, neurons, and potentially endothelial cells. Such adaptations should be tailored to the specific experimental context, particularly when the platform is used to address fundamental questions in glioma biology. A second limitation is the restricted characterization of the cellular composition of the spheroids. While the selected cell types were sufficient for this proof-of-principle study and for establishing the protocol, a more comprehensive analysis of the cellular composition (including the characterization of neurons and endothelial cells) and requirements for their long-term survival will be essential in future applications. This is particularly relevant for studies investigating drug effects on interactions of tumor and immune or neuronal cells. Moreover, the study lacks detailed molecular and cellular profiling and requires the implementation of viability and apoptosis assays as well as correlations to clinical outcomes in order to fully exploit the platform for broader basic and translational research questions.

In conclusion, we present a robust, cost-effective, and adaptable platform for the rapid generation of patient-derived glioblastoma spheroids that preserves key tumor-specific characteristics. This system enables reproducible, patient-specific functional analyses within a clinically relevant timeframe and provides a versatile foundation for translational applications, including drug response profiling and the evaluation of emerging therapeutic strategies.

## Figures and Tables

**Table 1:** Antibodies used for flow cytometry analysis.

Target	Dilution	Supplier	Catalog Number
CD14	1:20	BioLegend	325617
CD3	1:20	BioLegend	300440
CD45	1:20	BioLegend	304027
GFAP	1:400	Cell Signaling Technology	3655
IBA1	1:125	Proteintech	CL488-81728

**Table 2:** Antibodies used for immunohistochemistry analysis.

Target	Dilution	Supplier	Catalog Number
CD3ε	1:400	Cell Signaling Technology	85061
CD45RO	1:400	Cell Signaling Technology	55618
CoraLite® Plus 488 goat anti-mouse	1:500	Proteintech	RGAM002
CoraLite® Plus 555 goat anti-rabbit	1:500	Proteintech	RGAR003
GFAP	1:200	Cell Signaling Technology	3670
IBA1	1:800	Cell Signaling Technology	17198

## Data Availability

Any data collected within this study will be provided by the authors upon reasonable request.

## Supplementary Materials

The following supporting information can be downloaded at: https://www.mdpi.com/article/10.3390/cells15111021/s1, Figure S1: Immunohistochemistry images of spheroids from three different donors over time; Table S1: Clinical information on patient samples and their use in the study; Video S1: Formation of spheroids based on different cell numbers.

## Abbreviations

The following abbreviations are used in this manuscript:

IHC	Immunohistochemistry
GB	Glioblastoma
EGF	Epidermal Growth Factor
FGF	Fibroblast Growth Factor
FBS	Fetal Bovine Serum
MGMT	O6-methylguanine-DNA methyl-transferase
LDH	Lactate dehydrogenase
DMEM	Dulbecco’s Modified Eagle Medium
PBS	Phosphate-Buffered Saline
BSA	Bovine Serum Albumin
