# Supplementary material for: Generation of Heterotypic Primary Human Spheroids from Glioblastoma Resections and (Pre-)Clinical Applications
Source: Cells. 2026 Jun 2;15(11):1021. doi: 10.3390/cells15111021 (PMC13256787; doi:10.3390/cells15111021)
Supplement: Supplementary file 1 [file cells-15-01021-s001.zip › Figure S1.pdf]

specimen 6-CD45+CD3

Day	Hoechst	CD45	CD3	merge
6	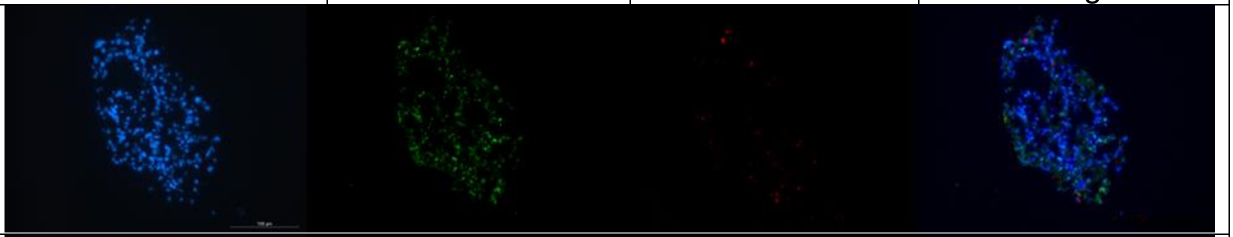			
7	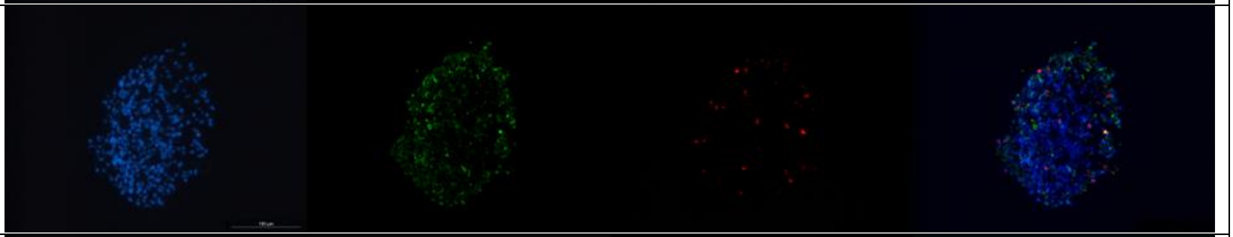			
9	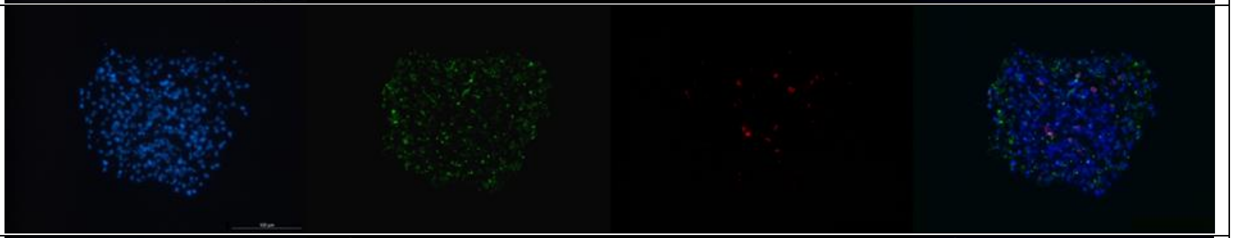			
12	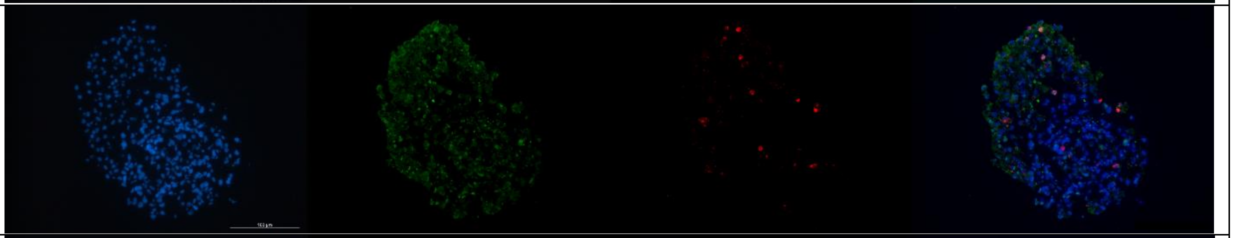			
14	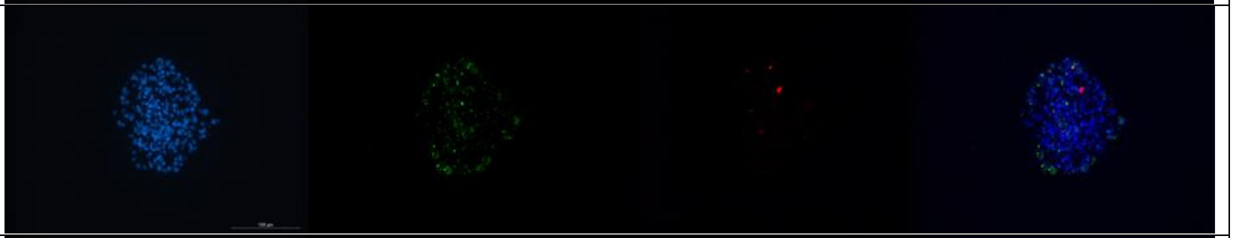			
16	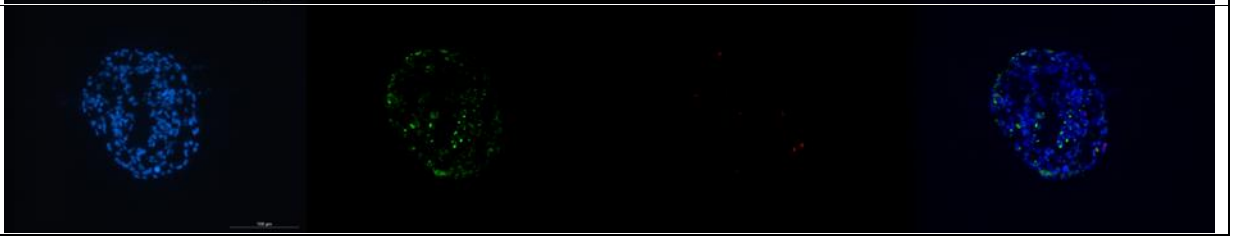			

specimen 6-GFAP+IBA1

Day	Hoechst	GFAP	IBA1	merge
6	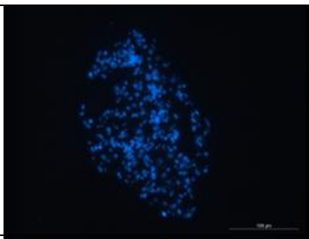	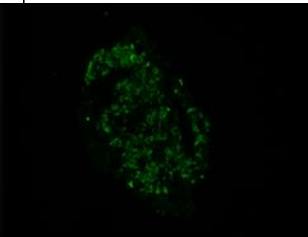	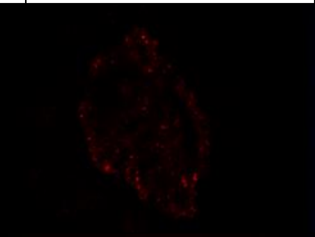	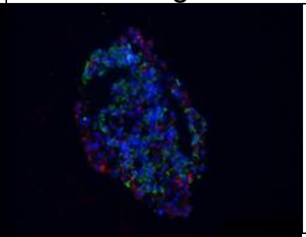
7	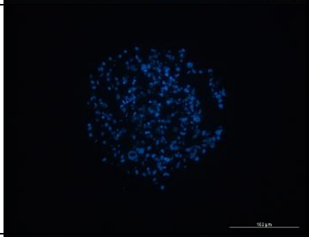	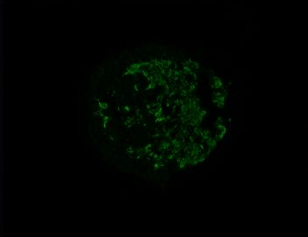	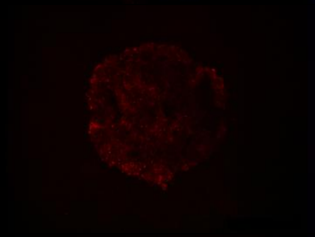	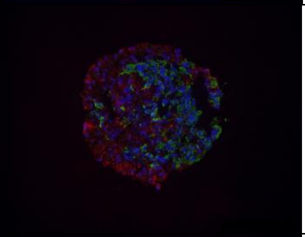
9	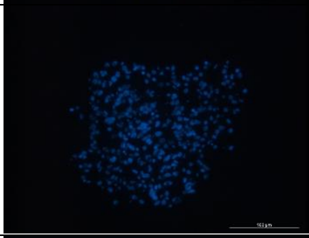	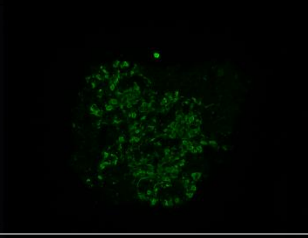	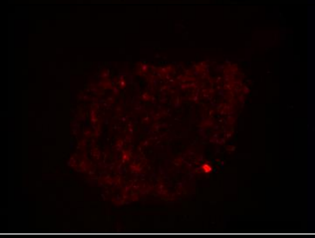	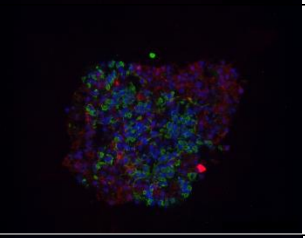
12	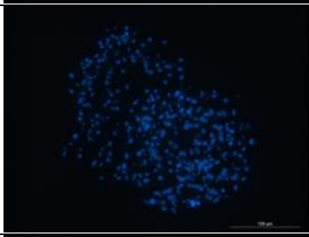	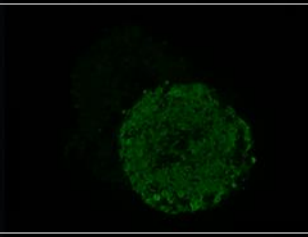	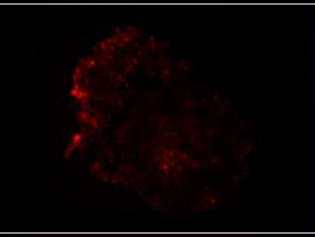	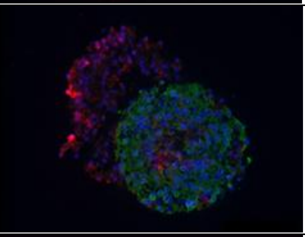
14	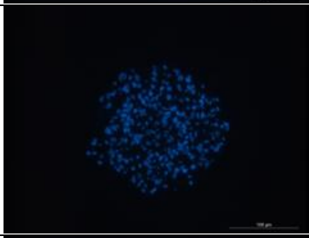	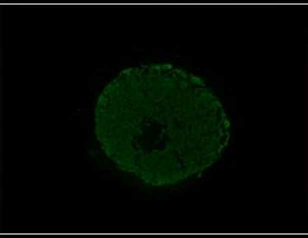	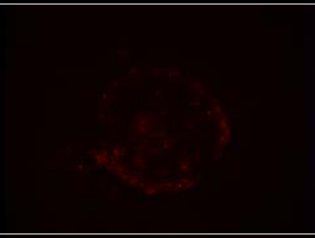	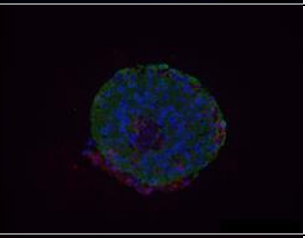
16	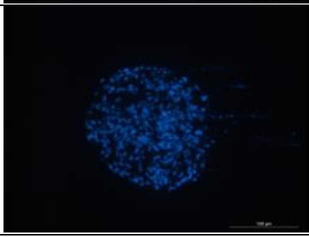	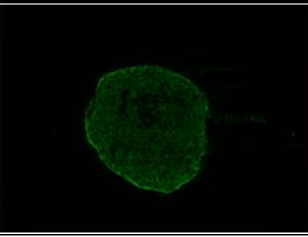	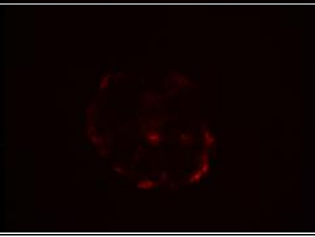	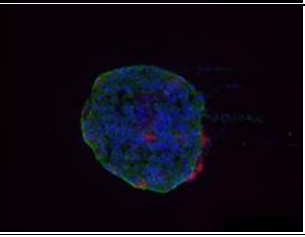

specimen 7-CD45+CD3

Day	Hoechst	CD45	CD3	merge
6	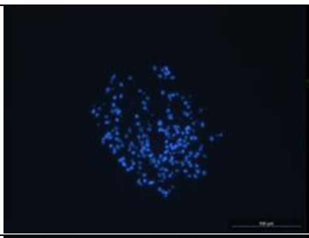	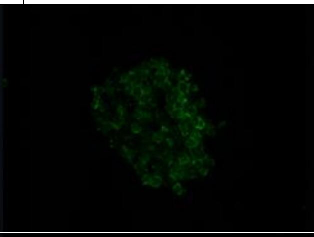	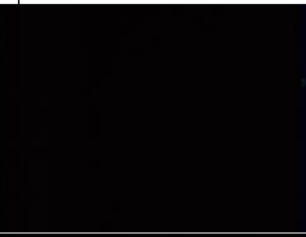	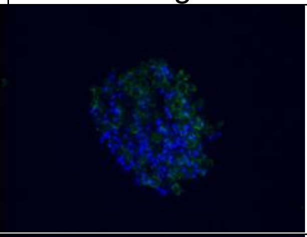
7	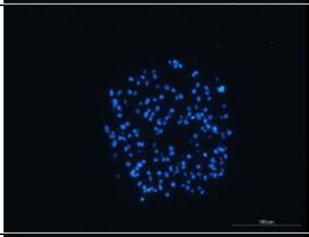	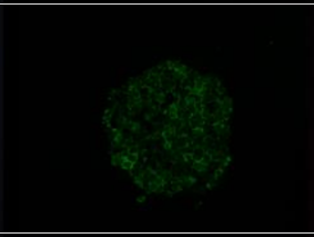	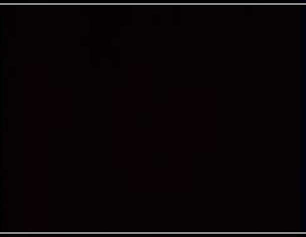	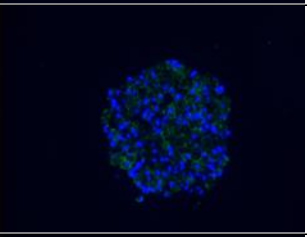
9	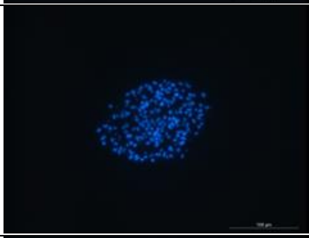	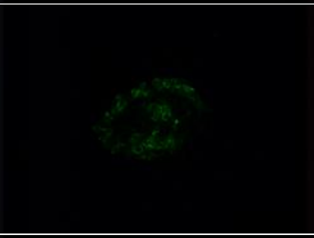	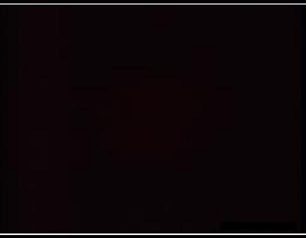	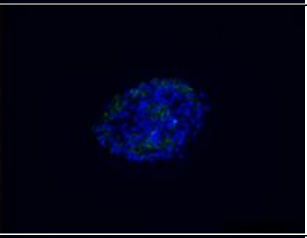
12	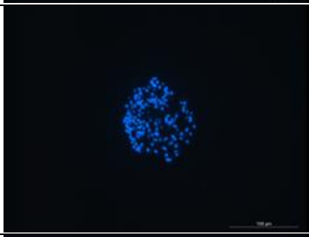	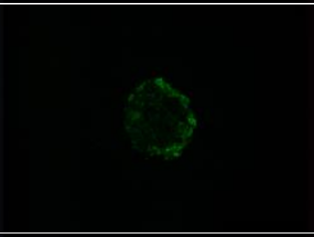	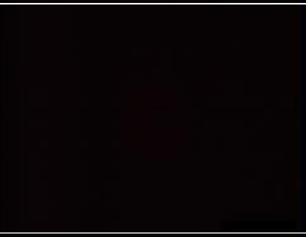	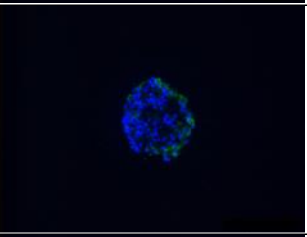
14	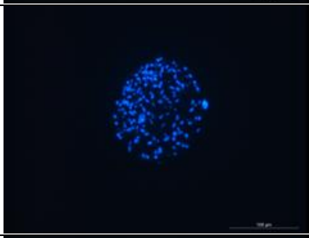	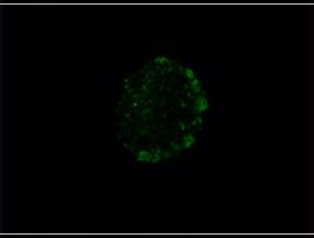	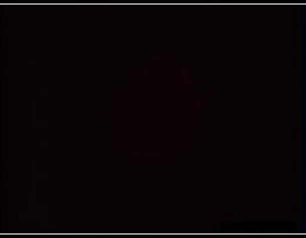	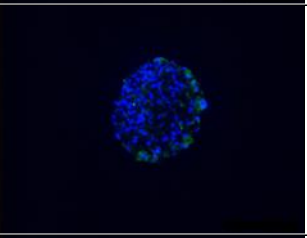
16	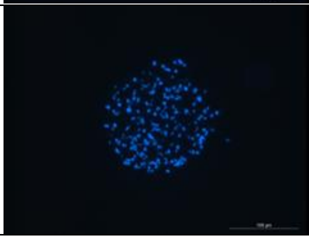	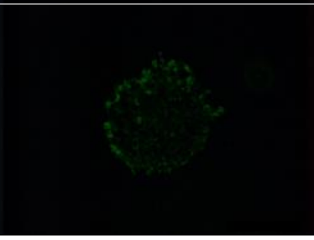	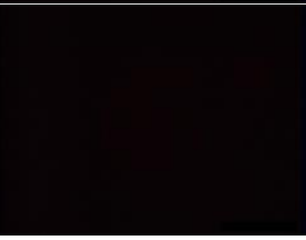	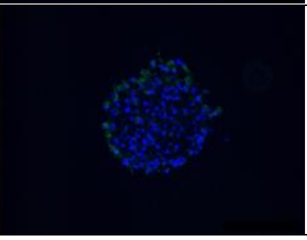

specimen 7-GFAP+IBA1

Day	Hoechst	GFAP	IBA1	merge
6	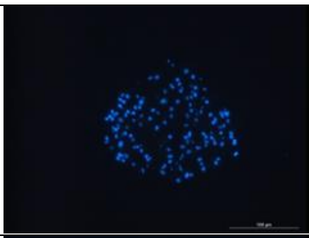	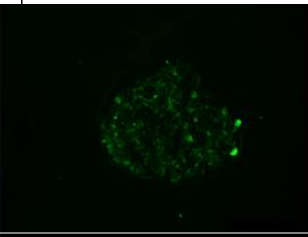	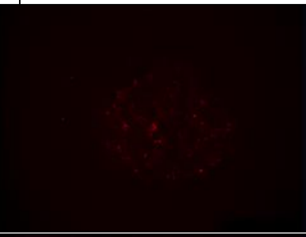	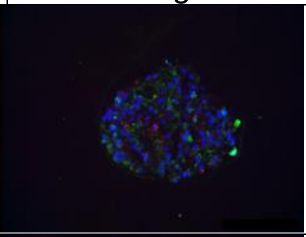
7	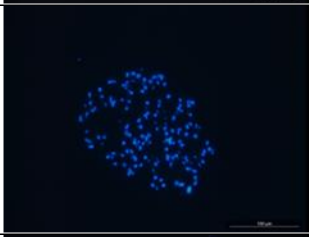	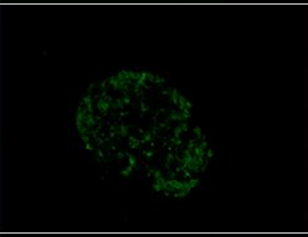	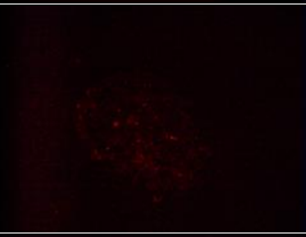	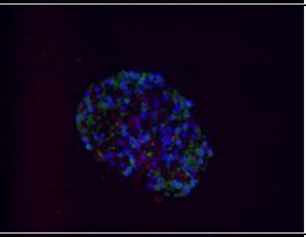
9	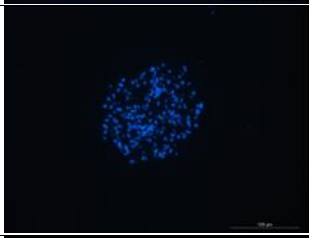	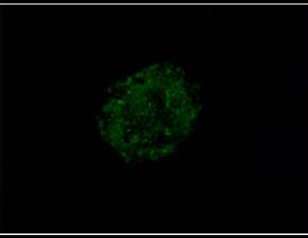	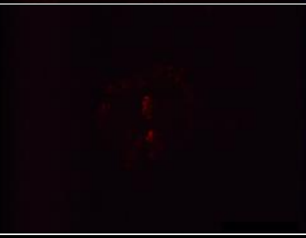	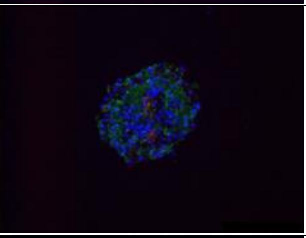
12	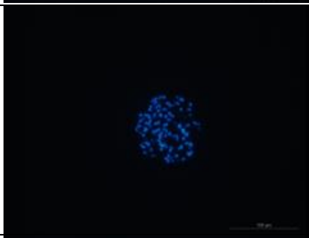	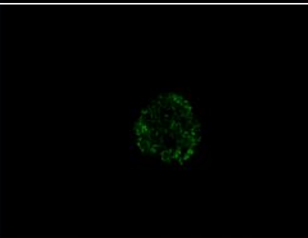	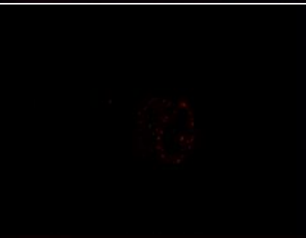	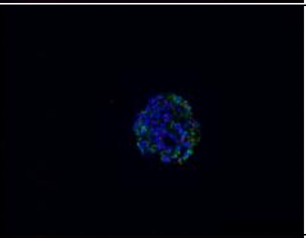
14	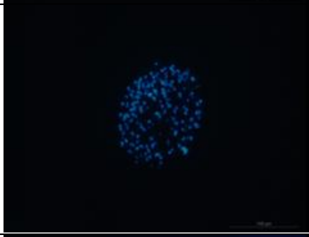	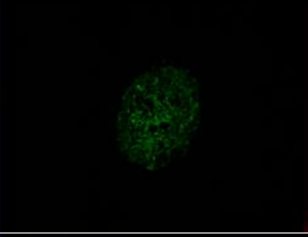	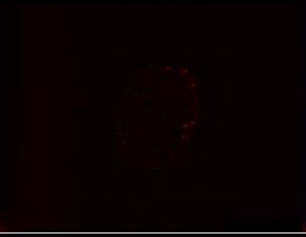	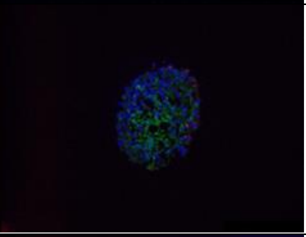
16	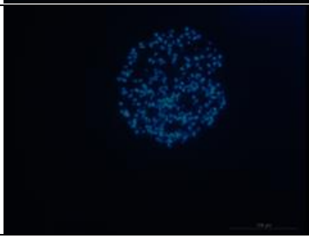	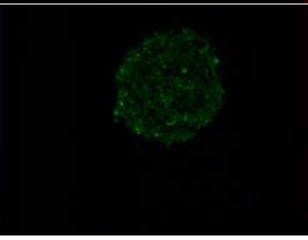	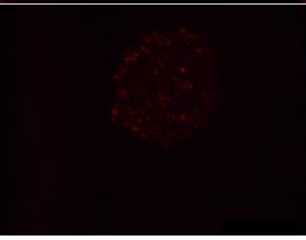	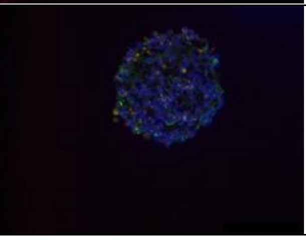

specimen 9-CD45+CD3

Day	Hoechst	CD45	CD3	merge
6	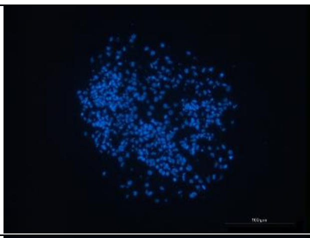	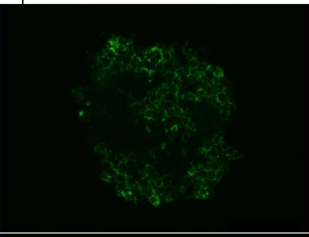	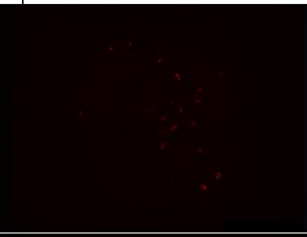	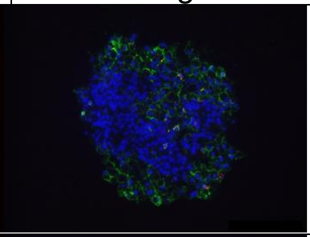
7	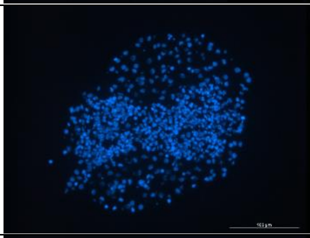	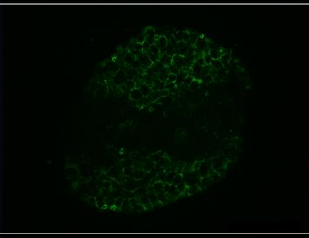	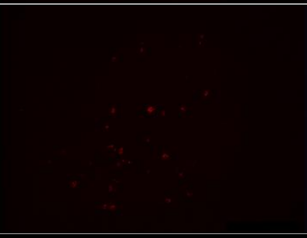	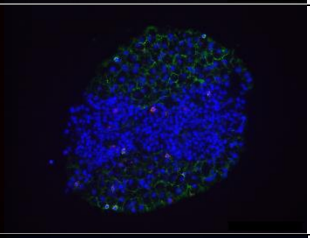
9	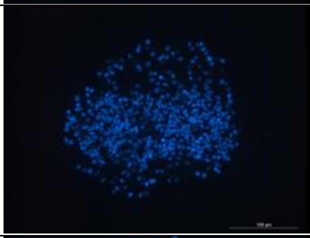	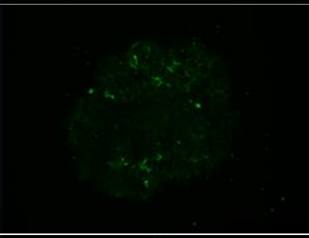	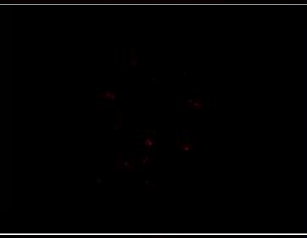	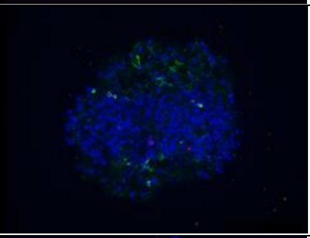
12	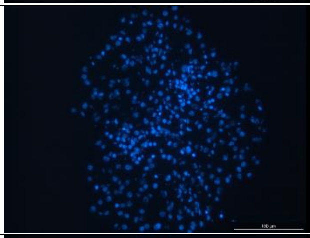	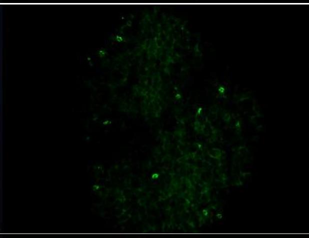	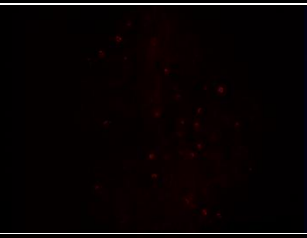	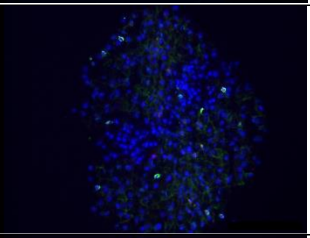
14	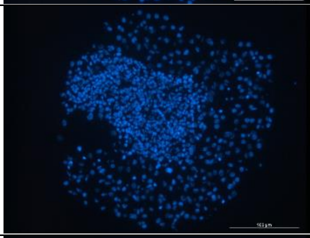	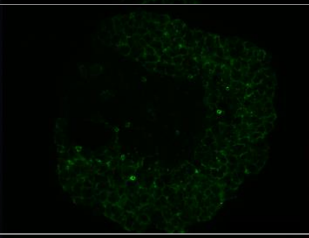	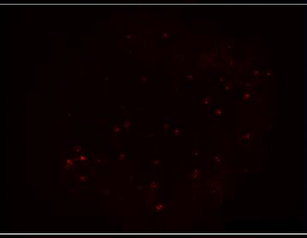	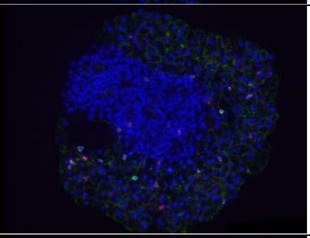
16	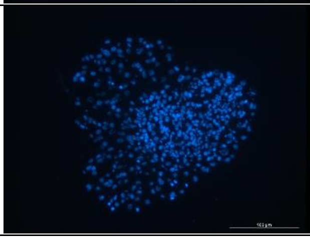	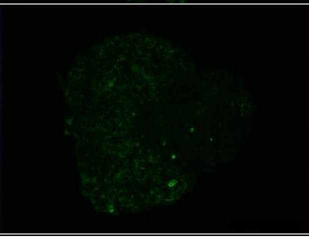	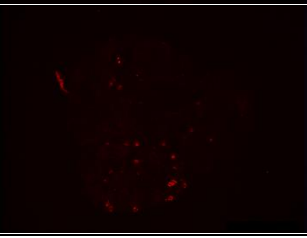	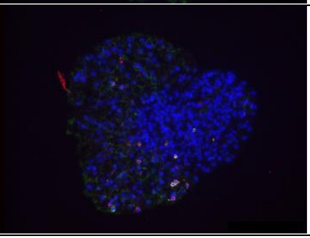

specimen 9-GFAP+IBA1

Day	Hoechst	GFAP	IBA1	merge
6	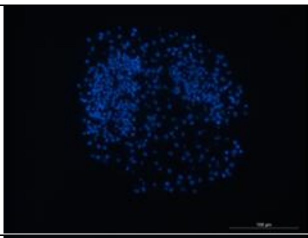	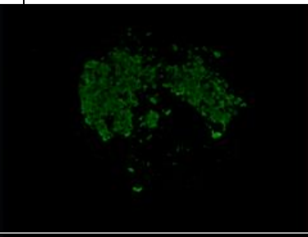	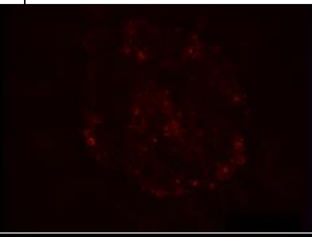	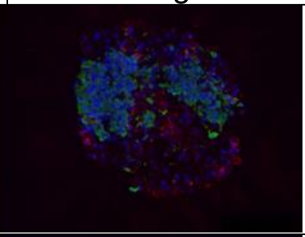
7	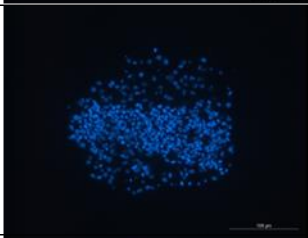	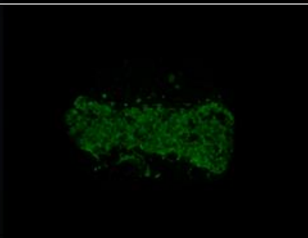	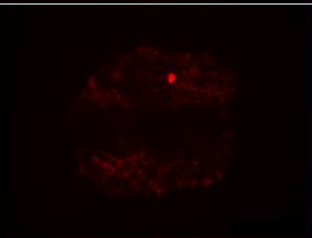	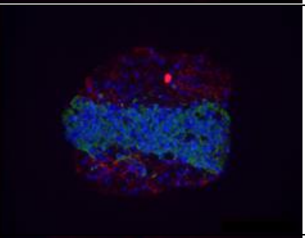
9	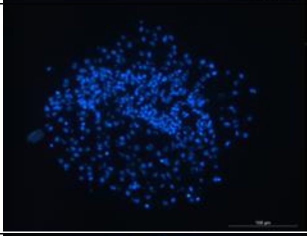	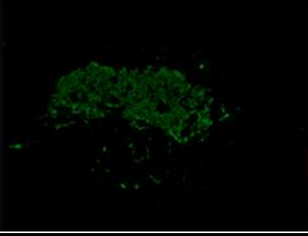	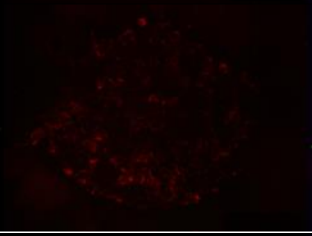	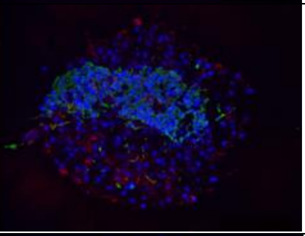
12	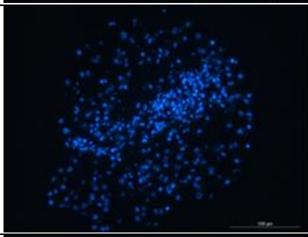	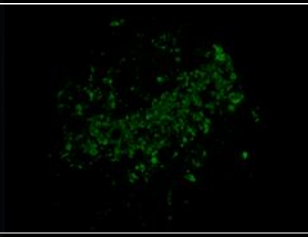	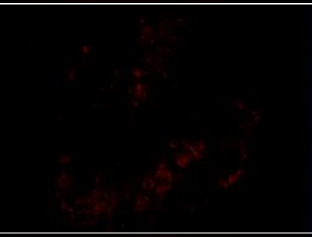	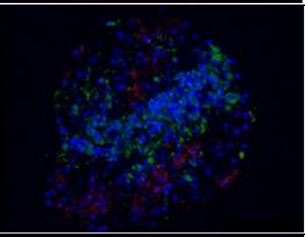
14	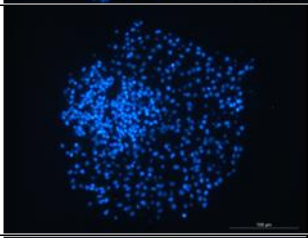	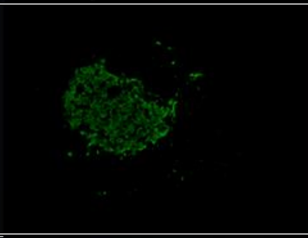	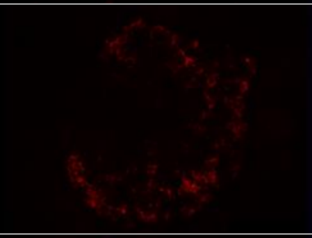	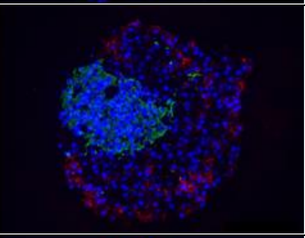
16	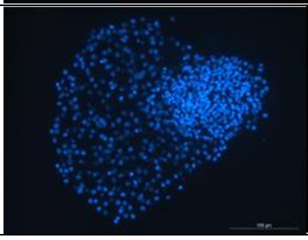	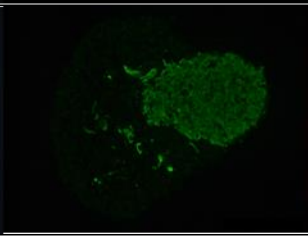	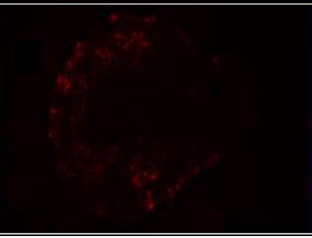	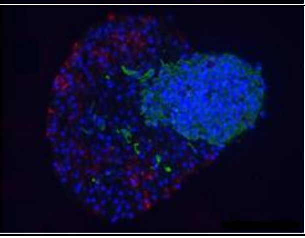
